# *In Vitro* Modelling of Respiratory Virus Infections in Human Airway Epithelial Cells – A Systematic Review

**DOI:** 10.3389/fimmu.2021.683002

**Published:** 2021-08-18

**Authors:** Laurine C. Rijsbergen, Laura L. A. van Dijk, Maarten F. M. Engel, Rory D. de Vries, Rik L. de Swart

**Affiliations:** ^1^Department of Viroscience, Postgraduate School of Molecular Medicine, Erasmus MC, University Medical Centre Rotterdam, Rotterdam, Netherlands; ^2^Medical Library, Erasmus MC, University Medical Centre Rotterdam, Rotterdam, Netherlands

**Keywords:** respiratory viral diseases, primary airway epithelium culture, organoid culture, co-culture, airway modeling

## Abstract

Respiratory tract infections (RTI) are a major cause of morbidity and mortality in humans. A large number of RTIs is caused by viruses, often resulting in more severe disease in infants, elderly and the immunocompromised. Upon viral infection, most individuals experience common cold-like symptoms associated with an upper RTI. However, in some cases a severe and sometimes life-threatening lower RTI may develop. Reproducible and scalable *in vitro* culture models that accurately reflect the human respiratory tract are needed to study interactions between respiratory viruses and the host, and to test novel therapeutic interventions. Multiple *in vitro* respiratory cell culture systems have been described, but the majority of these are based on immortalized cell lines. Although useful for studying certain aspects of viral infections, such monomorphic, unicellular systems fall short in creating an understanding of the processes that occur at an integrated tissue level. Novel *in vitro* models involving primary human airway epithelial cells and, more recently, human airway organoids, are now in use. In this review, we describe the evolution of *in vitro* cell culture systems and their characteristics in the context of viral RTIs, starting from advances after immortalized cell cultures to more recently developed organoid systems. Furthermore, we describe how these models are used in studying virus-host interactions, e.g. tropism and receptor studies as well as interactions with the innate immune system. Finally, we provide an outlook for future developments in this field, including co-factors that mimic the microenvironment in the respiratory tract.

## Introduction

Respiratory tract infections (RTIs) are a major source of morbidity and mortality in humans (1). Almost 300 million episodes of lower RTIs (e.g. pneumoniae and bronchitis) occurred in 2015 and about 3 million people die each year. This places RTIs amongst the leading causes of death worldwide (2, 3). A large fraction of RTIs is caused by viruses (50 to 90%) (4). Respiratory viruses include human rhinovirus (HRV), influenza A and B virus (IAV and IBV), human respiratory syncytial virus (HRSV), human metapneumovirus (HMPV), human coronavirus (HCoV), human parainfluenzavirus (HPIV), and human adenovirus (HAdV). Most respiratory viruses have single-stranded RNA genomes, with the exception of adenoviruses that have double-stranded DNA genomes. The highest morbidity and mortality due to RTIs is seen in infants, elderly and immunocompromised individuals, but also healthy individuals without underlying risk factors can be affected (5). Furthermore, RTIs have been associated with exacerbations of asthma or chronic obstructive pulmonary disease (COPD) (6, 7).

Recently, severe acute respiratory syndrome coronavirus 2 (SARS-CoV-2) has captured global headlines as the causative agent in the coronavirus disease 2019 (COVID-19) pandemic (8), highlighting the impact of respiratory viruses on global health. As this pandemic has shown, options for prophylaxis and treatment of viral respiratory infections are limited. For some viruses, such as influenza virus or adenovirus, vaccines are available but both efficacy and coverage are suboptimal. Antiviral drugs against acute respiratory virus infections often have limited efficacy. Therefore, development of novel and improved antiviral drugs and vaccines remains of high priority to improve global health.

The human respiratory tract (RT) is the primary site where respiratory viruses enter, replicate, disseminate and cause disease. These viruses are transmitted by aerosols and/or droplets. RTIs in most cases start by infection of airway epithelial cells in the upper respiratory tract (URT) (nasal cavity, pharynx, larynx), and are associated with common cold-like symptoms, including rhinitis, sore throat, runny nose, and nasal congestion. During the course of infection, the lower respiratory tract (LRT) (trachea, primary bronchi, lungs) can become involved, causing more severe disease, such as pneumonia or bronchitis (9). How severe lower RTIs develop and why only some individuals are affected could be a stochastic process related to dose and route of the inoculum. However, the development of severe LRTIs remains a black box and is an important topic of investigation. Many of these studies are performed *in vivo*, which provides important data on the pathogenesis of respiratory viruses [reviewed elsewhere (10–14)] but has some downsides, such as differences between animal or human host factors, ethical concerns and practical challenges. *In vitro* models have as advantages that they originate from the relevant host species, express the relevant host factors, are of less ethical concern and easier to work with than animal models. Therefore, a reproducible and scalable *in vitro* culture system that accurately represents the human RT would be valuable to study respiratory virus infections and test new treatments. Here we investigate the differences between commonly used *in vitro* primary airway cell culture models and how these models are used for studying respiratory virus infections. The first part of this review describes several characteristics of the RT, then we will review the evolution of *in vitro* cell culture systems in the context of viral RTIs, starting from advances after immortalized cell lines to recently developed stem cell (SC)-based organoid cultures. Finally, an outlook is provided for future developments in this field.

## Methodology and Study Design

This systematic review was prepared in accordance with PRISMA (Preferred Reporting Items for Systematic Reviews and Meta-analyses) guidelines (15). A systematic literature search, on the 21^st^ of January of 2021, was conducted using title, abstract, index term and author keyword fields for respiratory viruses and human airway epithelial cells (HAEC) or their variations. Of note, primary olfactory epithelial cells were not included, since there are described previously (16). Embase, Medline (Ovid), and Web of Science Core Collection databases (**Supplementary Material 1**) were searched from inception until 21^st^ January 2021. Search results were restricted to English and excluded case-reports. Articles were imported and deduplicated in EndNote™. Two researchers independently screened title and abstract for study eligibility criteria (17). Consensus was achieved after discussion upon disagreement. The electronic search was performed in Embase, Medline and WoS (**Supplementary Material 2**).

## Results

**Figure 1:**
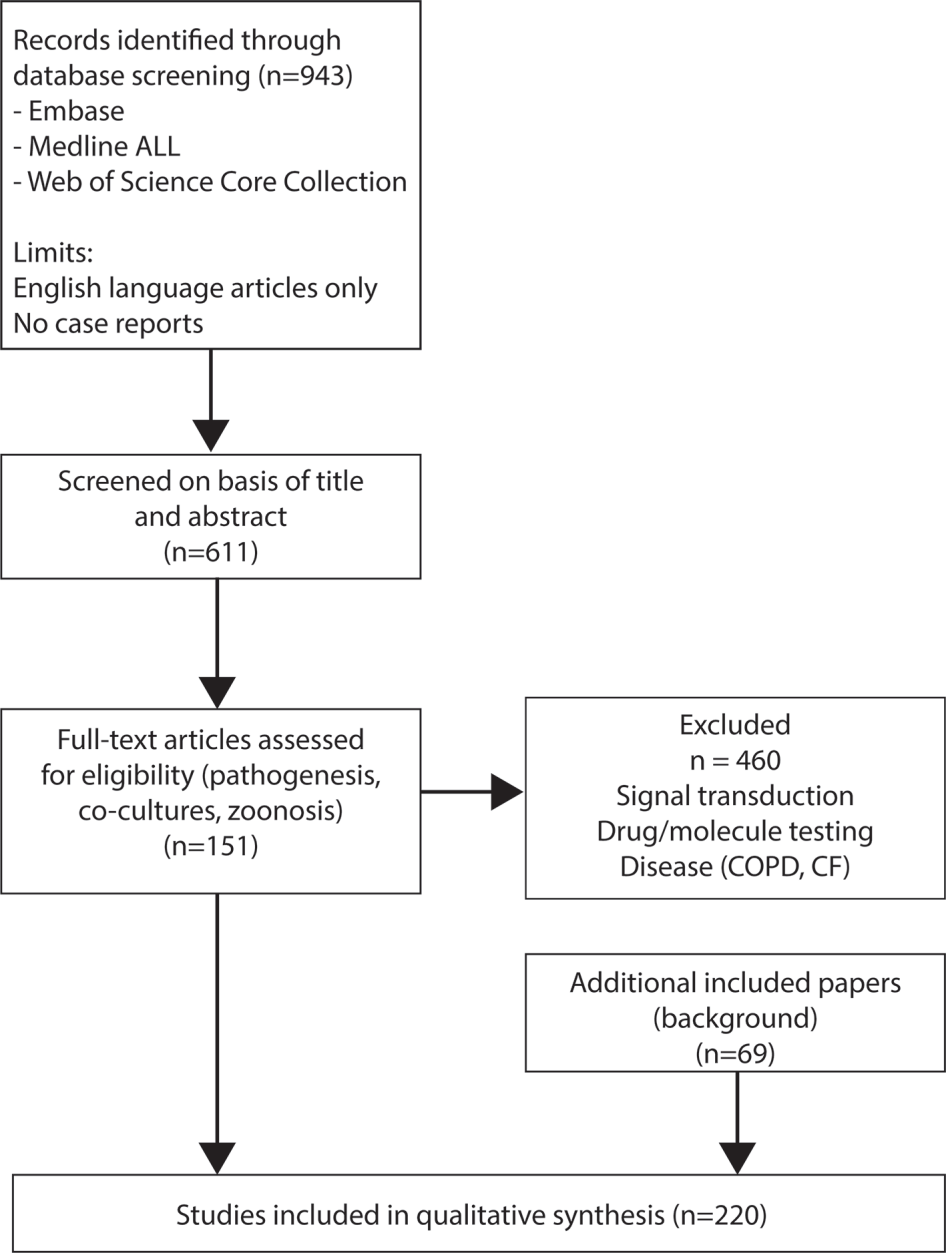
PRISMA flow diagram of the study selection process.

943 references were obtained and screened on basis of title and abstract (**Figure 1**). After the first screening, studies using only animal material, material of diseased patients, or immortalized cell lines were excluded. In addition, studies performed with respiratory bacteria (e.g. *Heamophilus influenzae*) and where adenoviruses were used as a vector were excluded. For the second screening full-text articles were assessed for eligibility and divided into seven topics: pathogenesis (N=165), signal transduction (N=93), co-cultures (N=21), zoonosis (N=11), reviews (N=32), drug/molecule testing (N=145), and disease (e.g. cystic fibrosis and chronic obstructive pulmonary disease) (N=28). Studies regarding signal transduction, drug/molecule testing and diseases such as chronic obstructive pulmonary disease and cystic fibrosis were excluded at this stage (n=504). Finally, 151 studies were selected for this systematic review. To provide sufficient background information we also included 69 additional papers based on relevant content for this review, resulting in a final selection of 220 papers.

## Discussion

### The Respiratory Tract

**Figure 2:**
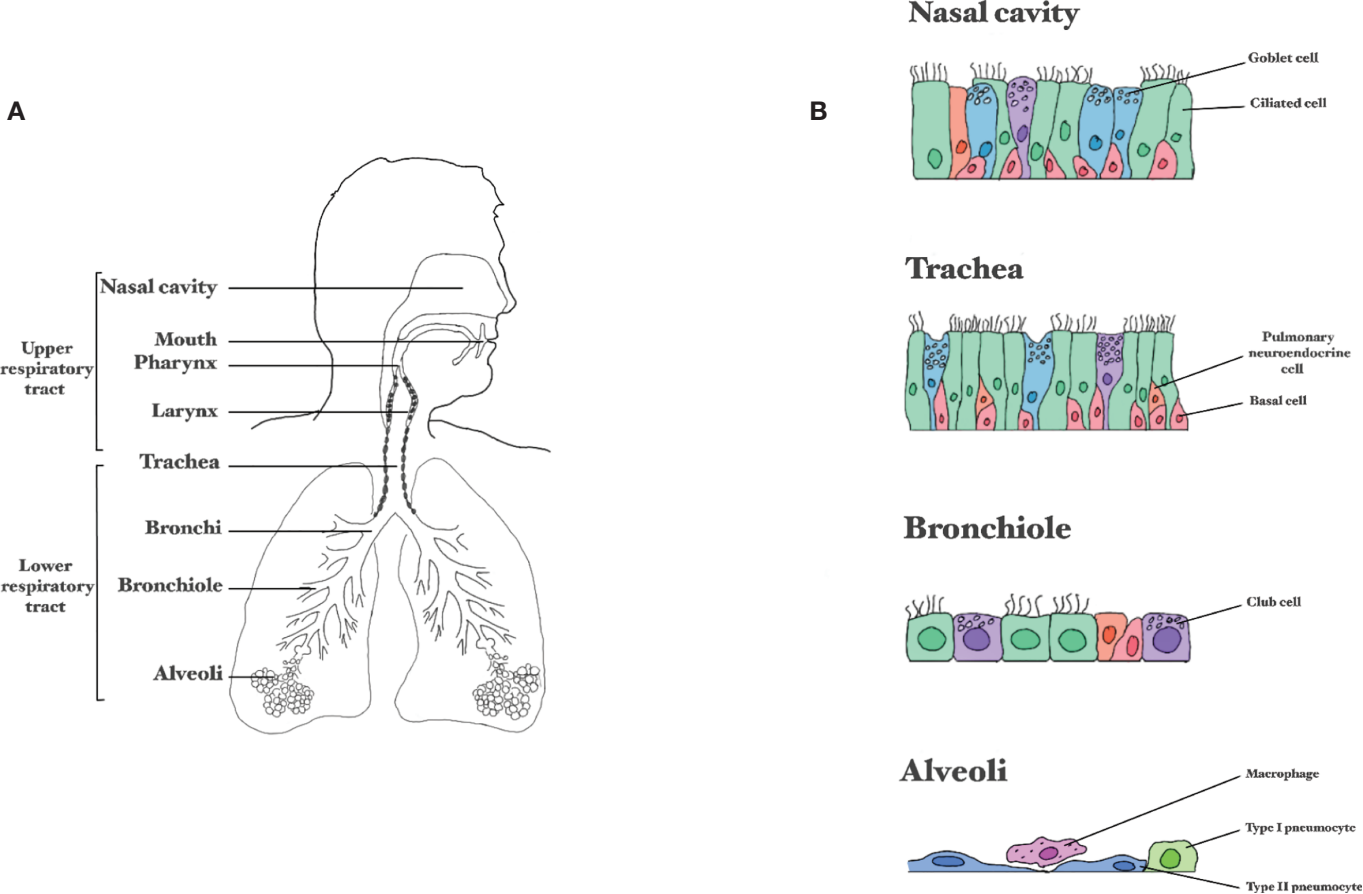
Composition of the respiratory tract. **(A)** Division between the upper and lower respiratory tract. **(B)** Schematic representation of epithelial layer in the different parts of the respiratory tract.

The RT can be divided into separate sections, either based on physiology (URT *versus* LRT, **Figure 2A**) or the type of respiratory epithelium (**Figure 2B**). The URT includes the nasal cavity, mouth, larynx and pharynx (throat), and beginning of the trachea (18). In the nasal cavity inspired air is warmed and humidified before it travels down the RT. Additionally, the nasal cavity houses the olfactory receptors that bind molecules resulting in impulses to the brain that enable smell. The nasal cavities drain into the nasopharynx that is in turn attached to the trachea (19). The trachea connects the throat to the LRT, starting at the primary bronchial branches (20, 21). The two bronchial branches bifurcate further into smaller tubes called the bronchi and bronchioles, which end in the alveoli; tiny air sacs facilitating gas exchange (22, 23). The entire RT is covered by epithelium and it is estimated that an adult of 176 cm or an infant of 60 cm have a lung epithelial surface of 78 m^2^ or 4 m^2^, respectively (24). The airway epithelium has a crucial barrier and immune functions. The mucus covering the airway epithelium is the first barrier that respiratory viruses have to pass to reach the airway epithelial cells. The airway epithelium itself has essential inflammatory, immune and regenerative capacities to combat these viruses.

The airway epithelium is composed of several cell types held together by tight junctions and adherens junctions that form a barrier against invading pathogens. The nasal cavity is lined with two distinct kinds of epithelium: the olfactory epithelium (1-2% of the nasal epithelium) and the respiratory epithelium (>98% of the nasal epithelium) (19). The neurons in the olfactory epithelium in the nose are directly connected to the central nervous system *via* the olfactory nerve. Viruses that infect the olfactory epithelium can use this nerve as a shortcut to reach the central nervous system (25). The epithelial layer in the nasal atrium consists of multi-layered keratinized squamous epithelium and further down the nose this becomes multi-row cylindrical epithelium. Many respiratory epithelial cells are also ciliated, facilitating mucus transport towards the pharynx and trachea (19). From the trachea downwards, the RT is lined with ciliated pseudostratified columnar epithelium (20). In the bronchioles, the epithelium gradually changes from pseudostratified into simple cuboidal and in the alveolar ducts and alveoli there is simple squamous epithelium (mainly type alveolar type I cells) (20).

Ciliated cells account for 50% to 80% of the airway epithelial cells, and have 200-300 motile cilia on their surface to displace mucus, enabling muco-ciliary clearance (26). The cells producing mucus are goblet cells, comprising around 9% of the respiratory epithelial cells (27). Next to goblet cells there are club cells; cuboidal non-ciliated non-mucous secretory cells. Club cells secrete extracellular matrix components, and can serve as progenitor cells for themselves and for ciliated cells (28). These cells are not very abundant in the URT, but in the terminal bronchioles account for approximately 11-22% of the cells (28). Epithelial and mucus-producing cells are supported by airway basal cells. Whereas the epithelial and mucus-producing cells are terminally differentiated and cannot renew, basal cells possess stem-cell like properties (29, 30). Basal cells occupy 31% of the respiratory epithelial cell population, the density of basal cells decreases when descending into the small airways (31). Another important cell type of the airways is the pulmonary neuroendocrine cell, comprising 1 out of 2500 epithelial cells from the trachea onto the alveolar ducts (32), forming the lung self-renewing SC niche relevant in airway epithelial regeneration (33). The alveolar epithelium consists mostly of two cell types, alveolar type I and type II cells (ATI and ATII). ATI are very thin, squamous cells, accounting for over 90% of the surface area in the lungs and provide an efficient barrier for air exchange (34). About 7% of the surface area comprises ATII, which are smaller and cuboidal, and function mostly in the production and uptake of lung surfactant (35). ATIIs can differentiate into ATI (36). In addition to ATI and ATII, also immune cells, like alveolar macrophages, are present in the alveoli.

Besides a barrier function, respiratory epithelial cells have inherent innate immunity functions. They recognize respiratory viruses via pattern recognition receptors, eventually leading to the production of cytokines and chemokines that render the cells and their neighboring cells in an antiviral state (37). The bridge between this innate response and adaptive immunity is formed by dendritic cells (DCs). There are tissue-resident DCs, that form an integrated network within the respiratory epithelium. Upon activation, for instance by a virus infection, they can travel to the lymph nodes to initiate an adaptive immune response. There are also migratory DCs that can be attracted to the site of infection and aid here in the local immune response. Besides DCs, natural killer cells, innate lymphoid cells, T cells and B cells orchestrate the immune response in the respiratory epithelium (38). Eventually, all elements contribute to an effective integrated immune response.

### *In Vitro* Models

Multiple *in vitro* respiratory cell culture systems have been developed to study the interaction between the different airway cell types and respiratory viruses. To date, the majority of these models is based on immortalized cell lines. Although useful in the study of direct viral infection and replication mechanisms at a cellular and molecular level, such monomorphic and unicellular systems fall short in creating an understanding of the processes that occur at an integrated tissue level (39, 40). We need more advanced models to better understand infection dynamics in relation to intrinsic cellular resistance mechanisms, including the innate immune response, and other factors such as microbiome and immune cells. New *in vitro* models have been proposed and used for some time, especially involving the use of primary HAEC. HAEC are derived from surgical material or brushings (nose or throat) that are subsequently cultured in the laboratory. More recently, 3D-cultures such as airway organoids (AO), formed from SCs, have been developed and hold promise as a useful tool to study host-pathogen interactions. However, this technique is still in its infancy, laborious and expensive. To perform in-depth studies into interactions between the host airway and respiratory viruses, the ultimate goal is to create a reproducible, scalable, feasible and economic *in vitro* culture system that faithfully recapitulates the architecture of the RT as well as the dynamics of infection.

#### Primary Respiratory Epithelial Cells

HAEC are obtained from human respiratory tissue, which can originate from different anatomical sites of the RT (41). Respiratory tissue is now obtained in different ways: during lung transplantation, during tissue resection in cancer patients, during other surgical procedures (e.g. turbinoplasty/-ectomy or nasal polypectomy) or from cadaveric explants (41). In addition, nasal and bronchial brushings, which are less invasive, can be performed to obtain HAEC (42). HAEC from healthy donors are now commercially available (43). Additionally, material from, for instance, COPD patients and/or smokers is also available (44, 45). Acquiring HAEC from various donors allows us to study and compare the RT of both healthy and diseased individuals.

#### Undifferentiated Primary Respiratory Epithelial Cells

Primary undifferentiated HAEC (HAEC_un_) are relatively easy to culture, but can only be passaged a few times. After obtaining tissue, the primary airway epithelial cells are directly isolated and cultured. For the isolation of primary airway epithelial cells from lung transplants or biopsies, the tissue is cut into smaller pieces and then dissociated *via* the addition of a protease-containing digestion cocktail, followed by generation of uniform single-cell suspensions (46, 47). Tissue obtained from brushings can be cultured directly (47). After isolation, HAEC can be used in this undifferentiated form for experiments. In addition to normal culture flasks or plates, HAEC can also be seeded on a collagen-coated semi-permeable membrane (transwell) (48). In this transwell system, medium is present on both the apical and the basolateral side. When a 100% confluent monolayer has formed to separate the apical and basolateral compartments, the HAEC_un_ can be used for experiments. These HAEC_un_ are not polarized and these cultures do not have ciliated cells or goblet cells, and therefore lack important characteristics of the airways.

#### Differentiated Primary Respiratory Epithelial Cells

Although more challenging to culture, differentiated HAEC (HAEC_dif_) represent the RT more faithfully than their undifferentiated counterparts. To differentiate primary respiratory epithelial cells in the transwell system, medium is removed from the apical compartment after the confluent monolayer has formed, creating an air-liquid interface (ALI). This, in combination with specific growth factors, induces differentiation of these cells over a timeframe of 3-4 weeks (48). Eventually, a polarized, pseudostratified respiratory epithelium is formed, containing basal cells, ciliated cells, and goblet cells or club cells (depending on the anatomical location) (48, 49). These HAEC_dif_ resemble the human RT anatomically (50, 51) but a continuous airflow, blood flow, and the presence of immune cells are still lacking.

#### Considerations for Use of Primary Cells

**Figure 3:**
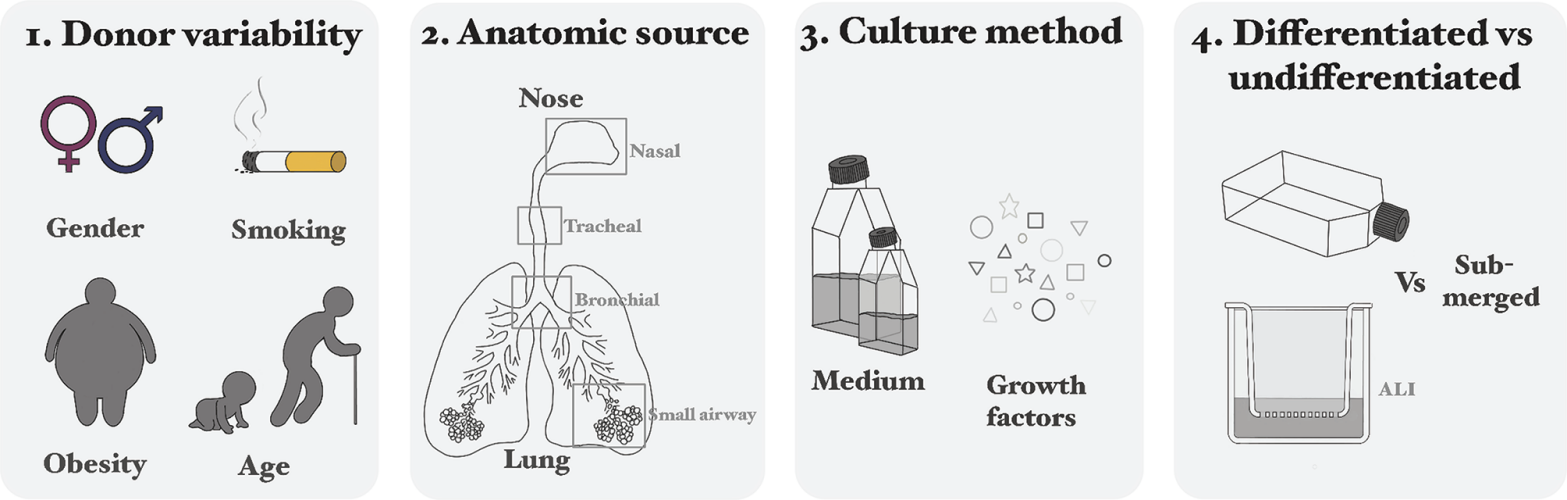
Considerations for using primary cells: differences in donor variability **(1)**, anatomic source of the cells **(2)**, culture methods (e.g. medium and growth factors) **(3)** and the use of undifferentiated *versus* differentiated cells **(4)**.

Comparing studies using primary HAEC is difficult, because of differences in donor variability **(1)**, anatomic source of the cells **(2)**, culture methods (e.g. medium and growth factors) **(3)** and the use of undifferentiated *versus* differentiated cells **(4)** (**Figure 3**).

1. Variability in culturability, morphology and phenotype between primary HAEC obtained from different donors has been described. This donor variability can depend on multiple factors, like age, gender, smoking history, or obesity (52–56). Variation in infection and replication levels of viruses between donors was regularly observed (57–60). Explanations could be differences in: receptor presence or distribution, cytokine responses, donors and environmental factors (61, 62). It is dependent on the research question how important these dissimilarities are.
2. In addition to donor variability, the source of the cells (ranging from nose to alveoli) can make comparison of studies difficult (63, 64), because of variations in susceptibility to viral infections and virus-host interactions (9). The preferential anatomical site should be determined by the research question (e.g. study URT or LRT) and comparing cells of multiple regions of the RT for susceptibility, virus replication and host responses.
3. The importance of culture medium is highlighted in a HRSV infection study, where two different media for differentiation of pediatric HAEC (nasal) were used: PneumaCult^TM^ and Promocell^TM^. The former led to an overall greater total number of cells, but the proportion of ciliated and goblet cells was similar. The replication kinetics of HRSV was similar in both cultures, but in the culture with PneumoCultTM more ciliated cells were infected. HRSV infection of the PneumoCultTM cultures led to a higher IFNλ secretion, which could be due to the increased infection of ciliated cells (65).
4. Several studies describe different expression patterns of cellular receptors that can mediate virus entry, resulting in varying infection percentages or different immune response between HAEC_un_ and HAEC_dif_ (66–69).

HAEC_un_ can be passaged approximately three times prior to differentiation, after which loss of epithelial integrity, differential gene expression, or senescence occurs (70–72). This restricts the number of experiments that can be performed. That is why immortalized variants of HAEC have been created. Jonsdottir and colleagues (2019) described that insertion of the Rho-associated protein kinase (ROCK) inhibitor Y-27632 *via* a lentiviral vector increased the longevity of the primary respiratory epithelial cells (73). Alternatively, exogenous induction of human telomerase reverse transcriptase (hTERT) can be used to prolong primary cell life (74). Immortalization resolves the issue of the restricted number of experiments, but it remains unknown if all the characteristics of the respiratory epithelial cells remain the same. And, even in these immortalized variants, senescence can occur after a few passages (75, 76). Genetic engineering of HAEC is challenging and may compromise translational aspects of the model.

In conclusion, primary HAEC can be extracted from all parts of the airways and differentiated into a pseudostratified monolayer resembling the *in vivo* situation. Although several variables and parameters must be considered, primary HAEC are a promising culture system to study RTIs.

#### Stem Cell-Based Models

SC-based models have the potential to overcome the problems associated with the limited lifespan of primary HAEC. SCs can be used to make organoids, which are three dimensional cultures that can self-organize and renew (77). Organoid culture is a novel and innovative technology and was first described in 2009 with organoids from the gut (78). In 2012, the technique to culture AO was developed (79). Until now, human AO are mostly made from SCs obtained from adult lungs. Adults tissues are more accessible than embryonic SCs or induced pluripotent (iPSCs) and have less ethical restraints. Nevertheless, AO from embryonic SCs are also used (80, 81). Although most studies describe AO obtained from the lung, AO can be made from almost all parts of the RT, and are thus a suitable alternative to primary HAEC (82). Additionally, genetic modifications are also more feasible in AO than in primary HAEC.

**Figure 4:**
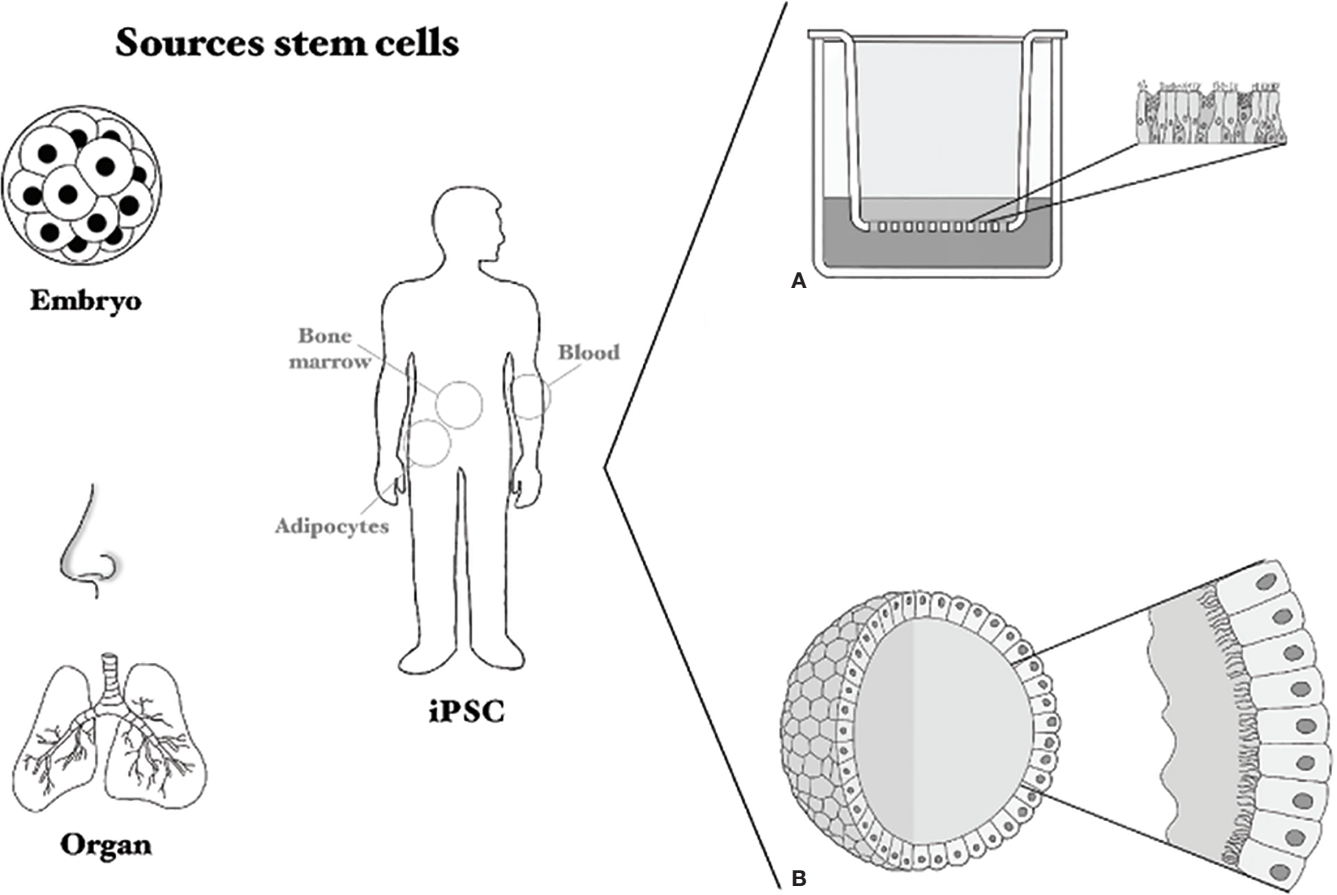
Sources of stem cells and culture methods. Several sources for stem cells are shown: embryonic stem cells, induced pluripotent stem cells (iPSC) and organ-derived or nasal brushing-derived. **(A)** Shows a Transwell system on which primary cells or stem-cell based cells can be cultured, **(B)** shows an airway organoid sphere with cilia and mucus on the inside.

Different SCs can be used to generate AO. First, embryonic SCs obtained from the inner cell mass of an early staged embryo have the potential to form every cell of the human body (83). However, the use of these cells remains ethically controversial. Second, iPSCs can also differentiate into almost every cell type of the human body (84). To obtain these iPSCs, somatic cells (for example skin cells) are reprogrammed to the progenitor state by addition of several transcription factors. However, specific factors, including growth factors and cytokines, are needed for differentiation into respiratory epithelial cells (85), which has so far not been successful. The challenges to find the optimal factors to differentiate these iPSCs into airway epithelial cells can be overcome by using organ-specific adult SCs. These cells can differentiate into the cell types of the respective organ (86). In this case, airway epithelial SCs can be isolated from tissue or URT brushings (87). The isolated basal cells can subsequently be used for the formation of 3D undifferentiated AO, using Matrigel and medium with specific growth factors. These AO, cultured either in 3D in matrigel or on transwell filters at ALI, can then be differentiated into cultures that represent the cells of the RT, such as basal cells, goblet cells, ciliated cells or alveolar cells (81, 87–92) (**Figure 4A**). In a differentiated spheroid AO, the basal cells are on the outside of the sphere, the goblet cells excrete their mucus into the lumen while the cilia move the mucus around (see **Figure 4B**). Similar to primary cell cultures, there are indications that culture medium influences organoid morphology and behavior, which is something that has to be studied in more detail (92, 93).

AO appear to be a promising model mimicking the human RT and once established can be maintained for a long period of time. Limited studies have been performed with AO and respiratory viruses so far due to the novelty of the technique. However, the studies that have been performed show great promise for studying virus-host interaction and drug screening studies in this model.

### Studying Respiratory Virus Infections

To understand the pathogenesis of respiratory virus infections, it is important to study (1) tropism, (2) receptor usage, (3) immune response, (4) cytopathic effects and damage, and (5) tissue regeneration after clearance. Primary cell culture models are crucial in these studies, because viruses rapidly adapt to culture in immortalized cell lines (81, 94, 95). In this second part of the review, we summarize how these properties of respiratory viruses have been investigated in light of the advantages of using primary HAEC and AO (**Table 1**).

**Table 1:** Summary table of the studies on respiratory viruses performed in primary respiratory epithelial cells and stem cell-based models.

Virus family	Characteristics (receptor, tropism, innate immune response)	Cell culture model	References
***Rhinovirus***			
**Receptor**	Major group: ICAM-1Minor group: LDL-RHRV-C CDHR3	HAEC_un/dif_ (bronchial and tracheal)	(96–101)
**Tropism**	Ciliated epithelial cells	HAEC_dif_ (bronchial)	(101)
**Cytokines**	TNF-α, IL-6, IL-8, and IP-10, IL-17C, type I IFN, type III IFN, CCL5, IL-1α, IL-1β, IL-1Ra, CXCL10	HAEC_dif_ (bronchial)	(102–104)
***Coronavirus***			
**Receptor**	HCoV-229E: CD13HCoV-HKU1: O-acetylated sialic acidSARS-CoV/SARS-CoV-2: ACE2, SXLMERS-CoV: DPP4	HAEC_dif_ (bronchial)Stem cell-based alveolospheres	(58, 66, 91, 105–108)
**Tropism**	Sars-CoV/SARS-CoV-2: ciliated epithelial cells, type III pneumocytes, club cellsMERS-CoV/HcoV-229: non-ciliated epithelial cellsHCoV-HKU1: Type I pneumocytes	HAEC_un/dif_ (bronchial)AOATIATIIStem cell-based alveolospheres	(57, 88–91, 105, 107, 109–111)
**Cytokines**	Type I IFN, type III IFN, CCL20, IL-33, CXCL5, CXCL6, CXCL8, CXCL20, IL-6, CXCL2, CXCL3, CXCL10, CXCL11, IL-17, IL-18, IL-1β, CCL4, CCL5, IL-8, IL-1α, IP-10, MIP-1β	HAEC_un/dif_ (bronchial)AOATIATIIStem cell-based alveolospheres	(90, 91, 110–114)
***Influenza virus***			
**Receptor**	Avian influenza viruses: α-2,3-linked oligosaccharidesHuman influenza viruses: α-2,6-linked oligosaccharides	HAEC_un/dif_AO	(93, 115)
**Tropism**	Ciliated cells, sometimes basal cells	HAEC_un/dif_ (nasal, bronchial, tracheobronchial)AO	(93, 115)
**Cytokines**	IL-1b, IL-6, IP-10, CCL2, CCL5, CXCL11, and IL-8, IL-1α, CXCL10, type I IFN, type III IFN	HAEC_un/dif_ (nasal, bronchial, tracheobronchial)	(116–118)
***Pneumovirus***			
**Receptor**	HRSV: CXCR3, IGFR1, nucleolin, HMPV: heparan sulfate	HAEC_dif_ (nasal and bronchial)	(119–121)
**Tropism**	Ciliated epithelial cells, type II pneumocytes, sometimes goblet cells and basal cells	HAEC_dif_ (nasal, tracheobronchial, bronchiolar)AO	(87, 122–125)
**Cytokines**	IL-6, IL-8, RANTES, type I IFN, type III IFN, CXCL-1, IP10, TFN-α, IL-10, CXCL-8, TRAIL, CXCL1	Primary (un)differentiated nasal and small airway epithelial cellshTERT-NECsAirway organoids	(126–132)
***Parainfluenza virus***			
**Receptor**	HPIV-1: α-2,3-linked sialic acid residuesHPIV-3: α-2,6-linked sialic acid residues	HAEC_dif_ (tracheobronchial)	(133–135)
**Tropism**	Ciliated epithelial cells	HAEC_dif_ (tracheobronchial)	(134, 136)
**Cytokines**	CXCL10 and CXCL11	HAEC_dif_ (tracheobronchial)	(137)
***Adenovirus***			
**Receptor**	HAdV-B type 14: Desmoglein 2HAdV-c type 5: CAR	HAEC_dif_ (bronchial)	(138)
**Tropism**	Lung parenchyma	AO	(139)
**Cytokines**	Meyer-Berg 2020 Stem cell research & therapy	HAEC_dif_ (bronchial)	(140)

HAECun, undifferentiated human airway epithelial cells; HAECdif, differentiated human airway epithelial cells; ATI, type I alveolar cells; ATII, type II alveolar cells; AO, airway organoids.

#### Receptor Studies

One of the mechanisms by which viruses enter a host cell is receptor-mediated entry; the appropriate receptor needs to be present on the cell for the virus to enter. Immortalized cell lines are often used to identify such a cellular receptor, for example by genomic- and interactome- based approaches (141, 142). Primary HAEC mimic the natural targets cells of respiratory viruses better than continuous cell lines and are thus important for receptor identification, as illustrated in the following examples.

For most HCoVs, receptors were initially identified in cell lines, followed by confirmation and verification of these receptors in primary HAEC (58, 66, 91, 105–107, 143–148). An example of the importance of using primary HAEC was seen in the search for a model for HCoV-HKU1. This virus only replicates in primary differentiated tracheobronchial epithelial cells. Through studies in these cells, the HCoV-HKU1 receptor (O-acetylated sialic acid) was ultimately identified (108, 149). Differential binding of avian and human influenza viruses to their receptors was investigated in primary HAEC_dif_ (bronchial), which express both α-2,3- and α-2,6-linked oligosaccharides. Infections with avian and human influenza viruses confirmed that viruses with avian origin preferably bind to α-2,3-linked oligosaccharides, whereas viruses from human origin bind to α-2,6-linked oligosaccharides (133). For most HRV subtypes their receptor was identified in immortalized cell lines and validated in HAEC, except for HRV-C (96–99, 150–153). For HRV-C the receptor was found in primary differentiated bronchial epithelial cells and in *ex vivo* organ transplants, since this HRV subtype does not replicate in continuous cell lines (101, 154).

Many HRSV receptor candidates have been proposed in literature, but arguably the relevant *in vivo* receptor has not yet been identified. Ciliated epithelial cells are the main target cell of HRSV (155), therefore primary HAEC_dif_ are the most suitable model to perform receptor studies. CXC3R1, insulin-like growth factor-1 (IGFR1) and nucleolin have been identified as potential cellular receptors for HRSV. CXC3R1 would probably not have been found if it were not for primary HAEC, since this receptor is absent (unless transfected) on immortalized cell lines (119, 120). The other two receptors have been confirmed and validated in primary HAEC (121, 156). Although closely related to HRSV, the proposed receptors for HMPV are not the same as for HRSV, but studies with primary respiratory epithelial cells from different locations in the RT have proposed integrins and heparin sulfate as receptors (126, 127, 157, 158).

Lastly, using primary HAEC_dif_ (tracheobronchial), it was confirmed that HPIV-3 uses α-2,6-linked sialic acid residues and HPIV-1 uses α-2,3-linked sialic acid residues as cellular receptors (133, 134). Primary HAEC have also been used to identify or verify the receptors for HAdV (138, 159).

All the above-mentioned examples clearly illustrate that HAEC are important for identifying the cellular entry receptor for respiratory viruses. Although initially the receptor is often found in cell lines, primary cell models are essential in confirming and validating this receptor and these results translate better to the *in vivo* situation.

#### Tropism

Regarding viral tropism, *in vivo* models and *ex vivo* culture models provide valuable information. However, it has to be kept in mind that in *in vivo* studies the animal is often not the natural host of the virus, introducing a possible bias. Human post-mortem material from either healthy or infected individuals is also useful for binding studies, but this material is not always available. Cell lines are immortalized and often express unique gene expression patterns that are not representative of *in vivo* cell types. Primary airway models can overcome these hurdles and are a thus powerful tool to investigate viral tropism, since most cell types from the RT are represented in these models.

In the SARS-CoV-2 pandemic, differentiated AO (AO_dif_) and primary HAEC rapidly helped elucidate that SARS-CoV-2 mainly infects ciliated cells, club cells and ATII, but not goblet cells (88–91, 109, 110). For other coronaviruses, similar models were used to determine their respective tropism, for instance HCoV-229E and MERS-CoV infect non-ciliated cells and HCoV-HKU1 predominantly infects ATII (105, 107, 160, 161). Multiple studies have been performed with influenza viruses in HAEC or AO. It was found that, for example, seasonal H1N1 was able to infect ciliated cells, goblet cells and alveolar cells, whereas pandemic H1N1 was also able to infect club cells (89, 93, 115). HRV is a common cause of respiratory infections in humans and both HRV-A and HRV-B can be cultured in conventional cell models, while this is not the case for HRV-C. However, by using 3D Matrigel cultures with primary HAEC_dif_ HRV-C can be propagated (159). For all three HRV subtypes the tropism could be determined in primary HAEC_dif_ (bronchial): HRV-A and HRV-B infect basal cells and HRV-C infects ciliated epithelial cells (101, 162). It was also confirmed that HRV can replicate in cells from both the URT (adenoids) and LRT (bronchus) (163). Clinical characteristics of HRV are also recapitulated in primary airway models. For instance, HRV-B infections are associated with less severe infections and studies in primary HAEC_dif_ (nasal and bronchial) reported that HRV-B replicates slower and leads to less cytotoxicity than HRV-A and HRV-C (164, 165). For both HRSV and HMPV it was confirmed in HAEC_dif_ and AO_dif_ mainly targets the ciliated epithelial cells (122, 123, 128, 166–168). One study showed that basal cells could also be infected by HRSV after epithelial injury, using commercially obtained primary HAEC_dif_ (bronchial) (124).

In conclusion, with primary airway cultures or SC-based cultures it is possible to culture viruses that are difficult to culture in immortalized cell lines and these models can provide reliable information on the viral tropism.

#### Disease Modelling

Studying pathogenesis is challenging *in vitro*, both in continuous and primary cell lines. A single or limited number of cell types are present in *in vitro* cultures, not resembling a full organism. As alternative, cytopathic effect (CPE) can be studied. CPE in primary HAEC_dif_ often resembles the natural situation more closely compared to continuous cells. HRSV offers a good example: in continuous cell lines it forms big syncytia (hence the name), but in primary HAEC HRSV forms little to no syncytia, which is in line with observations *in vivo* (both animal models and humans) (48, 122, 129, 168–170). A clinical observation in HRSV disease is neutrophil inflammation, which is involved in severe HRSV disease. This clinical phenomenon has been mimicked in HRSV-infected primary HAEC_dif_ (nasal) that were co-cultured with neutrophils (171). AO have significantly improved disease modelling opportunities. HRSV, HMPV and HPIV have been investigated in AO_dif_: and for example for HRSV epithelial cell sloughing was shown, which was absent in HPIV infections. These observations both fit with clinical observations. Other hallmarks of HRSV and HMPV disease, such as infection of ciliated epithelial cells and mucus production were also recapitulated in AO and in primary HAEC (80, 87, 125, 155, 172). These hallmarks are impossible to mimic in 2D cell lines and thus highlight the usefulness of 3D AO to study respiratory virus pathogenesis.

#### Innate Immune Responses

When a virus enters a cell, this cell will directly mount an innate immune response, both in the infected cell and in surrounding cells. This usually includes the production of several cytokines and chemokines to attract more immune cells and activate the adaptive immune system. Studying the innate immune system in primary HAEC has as an advantage over continuous cell lines, because they potentially produce more clinically relevant cytokines that would also be produced by the natural target cells. For instance, for SARS-CoV it was described that ATII are less susceptible than ATI, which was linked to a robust innate immune response (161). This pronounced innate immune response was not found in primary HAEC_dif_ (bronchial, chemically differentiated) after MERS-CoV, SARS-CoV or HCoV-229E infection, indicating immune evasion (173). It appears that each corona-virus induces specific innate immune responses, with few cytokines and ISGs overlapping (90, 91, 110, 112–114). In response to influenza virus infection, similar cytokines, mainly IL-1β, IL-6 and IL-8, were produced in HAEC_dif_ from different parts of the RT (114, 116–118). For HRSV and HMPV infections it has also been shown that similar cytokines, predominantly type I and/or type III IFN, are produced in HAEC_dif_ (nasal, bronchial and small airway) (123, 126–132, 168).

Different subtypes of HPIV are associated with certain clinical observations: HPIV1 can stay undetected for days, whereas HPIV2 is associated with mild disease and HPIV3 with more severe respiratory disease. These clinical observations have been linked to cytokine profiles that were found in primary HAEC_dif_ (tracheobronchial). It was shown that for HPIV1 there was no early innate immune response, whereas for HPIV2 innate cytokines were produced at early time points and for HPIV3 cytokines increased overtime (61).

It was observed that HRV induced an innate immune response in 3D matrigel cultures with primary HAEC_dif_ that inhibited viral replication, but that this was only short-lived because a second peak of viral replication was measured. This correlated with the production of the cytokines TNF-α, IL-6, Il-8, and IP-10. Based on these results, the authors postulated that the second peak appeared due to the production of new virus, after the eclipse caused by the innate immune system (102). Similar replication kinetics and cytokines were seen in other studies, including type I and type III IFN (103, 104, 174, 175). In one study it was shown that upon HRV infection IL-17C was produced in the basolateral compartment and induced CXLC1, which is a neutrophil attractant and may contribute to exacerbations of lower airway disease (103).

To summarize, many studies underline the importance of using primary HAEC and AO when studying innate immune responses. Although these models seem to be a good proxy for clinically relevant cytokines *in vivo*, studies directly comparing the innate cytokine responses between continuous cell lines and primary cell models are lacking. Nevertheless, complex cell systems that mimic the *in vivo* target cells of viruses probably model the antiviral innate immune response better than cell lines.

#### Other Applications of Primary Respiratory Epithelial Cells

Primary HAEC can mimic host factors related to lifestyle, such as smoking history and obesity. Smoking history might predispose to more severe SARS-CoV-2 disease, possibly to the upregulation of the ACE2 receptor, which was found in formalin-fixed paraffin-embedded human lung tissues and in brushings of the airway epithelium (176–178). This difference was not observed in primary HAEC_dif_ (bronchial), but the number of donors was limited (179). Obesity is related to more severe influenza symptoms, which has been shown in primary ATII, where cells from obese donors were more susceptible to a pandemic IAV than non-obese donors (52).

Primary HAEC are also useful for studying stability of viruses under environmental conditions. For example, primary HAEC_dif_ (bronchial) were used to study the impact of relative humidity on the stability of a pandemic influenza A virus in aerosols and droplets. Aerosols and droplets were created and supplemented with extracellular matrix material harvested from the apical surface of the HAEC and then the amount of virus was determined in MDKC cells (180). None of the experimental humidity conditions affected the stability of IAV H1N1 when aerosols and droplets were supplemented with extracellular matrix, but without supplementation, a humidity-dependent decay of virus infectivity was observed (180). This provided evidence that extracellular matrix can protect influenza viruses from decay and thereby promote spreading (181, 182).

Primary HAEC_dif_ have also been proposed as a screening model for evaluating and monitoring the infectivity, pathogenicity and antigenicity of virus variants, for instance during an outbreak. In the current SARS-CoV-2 pandemic many new, and supposedly more infectious, variants are arising. Information on these variants is important, so that interventions can be tailored. Using primary HAEC, it was found that SARS-CoV-2 variant (D614G) replicates better than the original wild-type strain in the nasal and proximal airways, which was not observed in VeroE6 cells. Increased replication in the URT might lead to more transmission (183). During influenza seasons of 2013-2014 and 2015-2016 the vaccine effectiveness against the circulating influenza strains was reduced. The reason for this was a reduction in sustained multi-cycle replication. This was found in primary HAEC_dif_ (nasal), but not in MDCK cells (182). These important data regarding novel virus variants would not have been available without primary airway models and are important for managing pandemics and developing interventions.

In this section, we illustrated that primary cell models may serve as an authentic model for *in vitro* respiratory viral pathogenesis studies, recapitulating viral infection in the host. They are useful for identifying viral receptors, determining viral tropism, mimicking disease and assessing innate immune responses in respiratory epithelial cells. Additionally, some viruses that are difficult to culture can be propagated in primary HAEC and often not in cell lines (184). Although continuous cell lines can give seminal insights into virus-host interactions, they also render selective pressure on viruses, leading to culture adaptations. One of the advantages of continuous cell lines is the possibility for genetic modification, which is thus far impossible in primary respiratory cell cultures. This hurdle can be overcome in the future by using AO derived from human SCs, which could be engineered to express desired host features.

### Co-Cultures

As described above, *in vitro* primary cell models can be used to investigate different aspects of virus-host interactions. However, still a big part of the micro-environment of the cells is not reflected in these models. (1) The lungs fill up with air and deflate during breathing, creating an airflow and stretching of the epithelium. (2) To supply the lungs with sufficient nutrients and to take up incoming oxygen, endothelial blood vessels are connected to the respiratory epithelial cells. (3) The airways are also normally colonized with all different kinds of micro-organisms, referred to as the microbiome. (4) Also, several types of immune cells are present to fight potential pathogens. These four features are usually not recapitulated in HAEs nor AOs, but are important for an integrated *in vitro* airway model.

#### Endothelial Cells

Endothelial blood vessels are connected to the airway epithelial cells *in vivo* and therefore important to also include in an *in vitro* airway model. Co-cultures with endothelium have been performed, with primary HAEC placed on a chip and microvascular endothelial cells on the opposite side of the porous membrane, with a fluid flow underneath (185). This small airway-on-a-chip model recapitulates tissue-tissue interactions, physicochemical microenvironments, and vascular perfusion of the RT (185, 186). Unfortunately, these models are costly and often not compatible with a biosafety level (BSL)-II or BSL-III laboratory environment required for performing pathogenic virus infections. A way to mimic air flow has been addressed by applying mechanical forces to the airway-on-a-chip model, recreating a breathing movement (187). In another study, primary HAEC were cultured at the apical side of transwell filters and primary microvascular endothelial cells at the basolateral side to create an alveolar-capillary system. This system was used to study influenza virus and staphylococcus aureus (SA) co-infections (188). Thus, although progress is being made, the use of transwell filters and airway-on-a-chip models to model the epithelial-endothelial barrier to study respiratory virus pathogenesis is still limited.

#### Microbiome

Next to co-cultures with endothelial cells, co-culture models with bacteria and/or viruses to mimic co-infections or the microbiome are being developed. One option is an infection with a bacterium preceding viral infection. In HAEC_dif_ (alveolar), co-cultured with primary microvascular endothelial cells, it was found that methicillin-resistant SA (MRSA) dysregulated the host immune response and decreased the barrier function (188). Another option is a viral infection preceding an infection with a bacterium, for instance initial IAV infection resulted in increased replication of *S. pneumonia* in primary HAEC_dif_ (bronchial). In contrast, in primary HAEC it was then found that pre-exposure to HRV reduced the viral titers of subsequent influenza virus exposure, which was supported by clinical data (189). Co-culturing cells with bacteria poses a challenge, since these micro-organisms replicate fast and rapidly overgrow the respiratory epithelial cells, often leading to cell death. In some of these studies, bacteria were inactivated to overcome this problem, but it is questionable if this still mimics the real-life situation since several features of the bacteria are lost (190). However, it has been shown that inactivated bacteria can influence respiratory virus infections. For instance, UV-inactivated *nontypable Heamophilus influenzae* (NTHI) enhanced the susceptibility of human bronchial epithelial cells to HPIV, likely due to upregulation of ICAM-1, a cellular receptor for HPIV (191, 192). Another way around the toxicity problem could be only co-culturing bacteria for a short time. Obviously, this does not mimic the microbiome or a co-infection (193–196). Nonetheless, this still allowed investigation of bacterial adherence to the (infected) primary HAEC or transmigration of bacteria. Alternatively, co-cultures could be regularly washed. Using this method researchers were able to culture NTHI with commercially available primary HAEC for 10 days (197). Combining co-cultures of bacteria and AO have, to our knowledge, not been described yet, but lessons can be learned from co-cultures of bacteria and intestinal organoids or skin organoids (197, 198).

It is important to study the effect of bacteria and viruses on the innate immune state of the HAEC. Certain innate immune profiles influence the outcome of viral infections, for instance for HRSV. It was described, in humans, that neutrophilic inflammation at the time of viral challenge predisposed individuals to symptomatic HRSV infection (199). These data suggest that co-cultures with, for instance, neutrophils might be required to fully mimic the microenvironment of the RT. In conclusion, although there is only limited data available on this topic, more studies are being performed and we are confident it is feasible to create an *in vitro* airway model combined with the microbiome.

#### Immune Cells

Another important feature of the airways is the (intra-epithelial) presence of immune cells that are often involved in the defense against respiratory virus infections. Co-cultures of neutrophils with primary HAEC_un_ (trachea) that were infected with HPIV were already performed in the 1990s (200, 201). These studies showed enhanced adhesion of neutrophils to HPIV-infected cells. This has later been shown in AO infected with HRSV, where it was even possible to visualize the preferential neutrophil movement to the infected AO. Co-cultures of primary HAEC (nasal or bronchial) with DCs or T cells have also been described, either with virus present prior to adding the cells, or after. In these studies, researchers investigated both the effect on the immune responses as well as viral replication (87, 202, 203). They showed that the presence of DCs or T cells enhanced antiviral and inflammatory responses and inhibited viral replication. A triple co-culture has also been described, in which primary HAEC_dif_ (bronchial) were co-cultured with monocyte-derived DCs (moDCs) and macrophages (204). Here, the moDCs were infected with HRSV and added apically to a layer of HAEC in a transwell system. Uninfected moDCs were simultaneously added to the basolateral membrane of the insert. This led to the transmission of HRSV to the HAEC, but not to the moDCs located basolaterally. However, when macrophages were added to the apical surface of this co-culture, the basolateral moDCs were infected too, indicating some type of trans-epithelial transport mechanism. This study shows the importance of having multiple factors in a culture to understand the infection process (205). All in all, combining primary HAEC_dif_ with endothelial cells, airflow, the microbiome and immune cells would be an ideal model to mimic our RT.

## Conclusion and Future Directions

We need scalable, feasible, affordable and reproducible *in vitro* models that adequately reflect the complexity of our RT and the cascade of events that occur during viral infections. We illustrated in this review that primary HAEC and AO are promising models for the RT and for studying respiratory viral pathogenesis. Since the field of primary epithelial cell models and AO is rapidly progressing, future applications of these models are endless. Currently these models are used for testing and developing effective treatments for SARS-CoV-2 (206). Another potential future application of primary HAEC is using species-specific cells to study the zoonotic potential of viruses. With these models it is possible to test the infectivity of the viruses isolated from the natural hosts in primary human respiratory epithelial cells and vice versa (207–210).

Furthermore, primary HAEC and AO can be used for drug screening studies, to test the potential of a drug, small molecule or antibody to inhibit virus replication in the context of a more representative tissue environment. There are many papers describing drug studies performed in HAEC (211). In the future, the use of AO for drug screening studies can be of great value as well (212).

The ultimate goal is to design a model that reflects the respiratory environment in all critical aspects to understand respiratory virus infections and host interaction in detail. Therefore, the usage of self-organized AO models would be preferable over the use of primary HAEC, either in 2D at ALI or 3D. Although initially more expensive and laborious to set up, such AO cultures can be maintained for long periods and are expected to generate the most reproducible results. However, primary HAEC are still a good model to use, especially for rapid screening of inter-individual variation of different donors in response to respiratory virus infections, or fast initial drug screening.

A state-of-the-art development is the lung-on-a-chip model. This model allows co-culture of primary respiratory epithelial cells, for instance primary HAEC or AO, with endothelial cells and mimics breathing by using an airflow and stretching. This model might recapitulate the human airways most faithfully, but there are still many challenges to be overcome before this can be used widely. This model is expensive, and requires specific expertise and equipment. One of the biggest hurdles is using the lung-on-a-chip model in a BSL-II or higher laboratory environment. However, the first study using a lung-on-a-chip model in combination with a respiratory virus (Influenza A) has been published recently showing the potential of this model (213).

In conclusion, primary HAEC are better suited for investigating basic virus-host interactions, such as receptor use and tropism of a respiratory virus than cell lines. However, for investigating the more complex interplay between virus, target cells and immune responses, AO appear to be more suitable. Further improving this model by introducing additional system factors, such endothelium and airflow (as has been done for lung-on-a-chip models), commensal bacteria and immune cells are required to even more closely mimic the micro-environment present in the RT. This culture system will help us understand viral respiratory infections and host responses, and to develop effective therapies to cure these infections.

## Data Availability Statement

The original contributions presented in the study are included in the article/**Supplementary Material**. Further inquiries can be directed to the corresponding author.

## Author Contributions

LR, LD, RV, and RS have been involved in conceptualization, project administration, and visualization. ME was responsible for the methodology with direct involvement of LR and LD. LR and LD were responsible for data curation, investigation, formal analysis, and wrote the original manuscript. All authors contributed to the article and approved the submitted version. RV and RS have been responsible for the supervision. In addition to RV and RS, ME was also involved in providing the resources.

## Funding

Funds were received for open access publication by the Erasmus University Library Data and Publication support.

## Conflict of Interest

The authors declare that the research was conducted in the absence of any commercial or financial relationships that could be construed as a potential conflict of interest.

## Publisher’s Note

All claims expressed in this article are solely those of the authors and do not necessarily represent those of their affiliated organizations, or those of the publisher, the editors and the reviewers. Any product that may be evaluated in this article, or claim that may be made by its manufacturer, is not guaranteed or endorsed by the publisher.

## Supplementary Material

Supplementary material 1 Core collection Erasmus MC.

Supplementary material 2 Electronic search terms for Embase, Medline and Web of Science.
