# Supplementary material for: In Vitro Modelling of Respiratory Virus Infections in Human Airway Epithelial Cells – A Systematic Review
Source: Front Immunol. 2021 Aug 18;12:683002. doi: 10.3389/fimmu.2021.683002 (PMC8418200; doi:10.3389/fimmu.2021.683002)
Supplement: Supplementary material 1 — Core collection Erasmus MC. [file DataSheet_1.docx]

*Supplementary material 1: Core collection Erasmus MC*

Science Citation Index Expanded (1975-present) ; Social Sciences Citation Index (1975-present) ; Arts & Humanities Citation Index (1975-present) ; Conference Proceedings Citation Index- Science (1990-present) ; Conference Proceedings Citation Index- Social Science & Humanities (1990-present) ; Emerging Sources Citation Index (2015-present)
