# Supplementary material for: In Vitro Modelling of Respiratory Virus Infections in Human Airway Epithelial Cells – A Systematic Review
Source: Front Immunol. 2021 Aug 18;12:683002. doi: 10.3389/fimmu.2021.683002 (PMC8418200; doi:10.3389/fimmu.2021.683002)
Supplement: Supplementary material 2 — Electronic search terms for Embase, Medline and Web of Science. [file DataSheet_2.docx]

*Supplementary material 2: Electronic search terms for Embase, Medline and Web of Science*

Embase

('respiratory virus'/de OR 'Coronavirinae'/de OR 'influenza'/exp OR 'Pneumovirinae'/de OR 'Rhinovirus'/de OR 'Adenoviridae'/exp OR 'Respirovirus'/de OR 'viral respiratory tract infection'/de OR 'Parainfluenza virus infection'/de OR 'respiratory syncytial virus infection'/de OR 'Influenza virus'/exp OR 'Pneumovirinae'/exp OR ('respiratory tract infection'/exp AND 'virus infection'/de) OR (coronavirus* OR influenza* OR pneumovir* OR metapneumovir* OR rhinovirus* OR adenovir* OR parainfluenz* OR respirovir* OR ((respirator* OR respiro* OR lung* OR corona* OR pneumo* OR metapneum* OR rhino* OR adeno* OR pulmon*) NEAR/3 (virus* OR viral))):ab,ti,kw) AND ((('primary cell'/de OR 'primary cell culture'/de OR 'organoid'/de) AND ('respiratory epithelium'/exp OR 'lung alveolus cell'/exp)) OR (((respirator* OR respiro* OR lung* OR pneumo* OR pulmonar* OR bronch* OR airway* OR nose* OR nasal* OR trach* OR larynx* OR alveol*) NEAR/3 (organoid* OR primary-human* OR cell-isolat* OR tissue-isolat*)) OR ((respirator* OR respiro* OR lung* OR pneumo* OR pulmonar* OR bronch* OR airway* OR nose* OR trach* OR larynx* OR alveol*) NEAR/3 (primar*) NEAR/3 (cell* OR cyte* OR culture* OR epithel*)) OR ((primar*) NEAR/3 (pneumocyt* OR AEC* OR HAE OR HAEs OR HAEC* OR NHBE* OR wdNHBE* OR BEC OR NHNC OR NEC OR hNEC)) OR PAEC* OR PBEC* OR HNEpC):ab,ti,kw) NOT ((animal/exp OR animal*:de OR nonhuman/de) NOT ('human'/exp)) NOT ([Conference Abstract]/lim) AND [ENGLISH]/lim NOT ('case report'/exp OR (case-report*):ti)

Medline

(exp Coronaviridae/ OR Influenza, Human/ OR exp Pneumovirinae/ OR Rhinovirus/ OR exp Adenoviridae/ OR exp Respirovirus/ OR exp Paramyxoviridae Infections/ OR exp Influenzavirus A/ OR exp Influenzavirus B/ OR exp Influenzavirus C/ OR exp Paramyxoviridae/ OR (respiratory tract infection/ AND virus infection/) OR (coronavirus* OR influenza* OR pneumovir* OR metapneumovir* OR rhinovirus* OR adenovir* OR parainfluenz* OR respirovir* OR ((respirator* OR respiro* OR lung* OR corona* OR pneumo* OR metapneum* OR rhino* OR adeno* OR pulmon*) ADJ3 (virus* OR viral))).ab,ti,kf.) AND (((Primary Cell Culture/ OR Organoids/) AND (exp Respiratory Mucosa/ OR exp Pulmonary Alveoli/)) OR (((respirator* OR respiro* OR lung* OR pneumo* OR pulmonar* OR bronch* OR airway* OR nose* OR nasal* OR trach* OR larynx* OR alveol*) ADJ3 (organoid* OR primary-human* OR cell-isolat* OR tissue-isolat*)) OR ((respirator* OR respiro* OR lung* OR pneumo* OR pulmonar* OR bronch* OR airway* OR nose* OR trach* OR larynx* OR alveol*) ADJ3 (primar*) ADJ3 (cell* OR cyte* OR culture* OR epithel*)) OR ((primar*) ADJ3 (pneumocyt* OR AEC* OR HAE OR HAEs OR HAEC* OR NHBE* OR wdNHBE* OR BEC OR NHNC OR NEC OR hNEC)) OR PAEC* OR PBEC* OR HNEpC).ab,ti,kf.) NOT (exp Animals/ NOT Humans/) NOT (news OR congres* OR abstract* OR book* OR chapter* OR dissertation abstract*).pt. AND english.la. NOT (Case Reports/ OR (case-report*).ti.)

Web of Science

TS=((("respiratory tract infection"/exp AND "virus infection"/de) OR (coronavirus* OR influenza* OR pneumovir* OR metapneumovir* OR rhinovirus* OR adenovir* OR parainfluenz* OR respirovir* OR ((respirator* OR respiro* OR lung* OR corona* OR pneumo* OR metapneum* OR rhino* OR adeno* OR pulmon*) NEAR/2 (virus* OR viral)))) AND ((((respirator* OR respiro* OR lung* OR pneumo* OR pulmonar* OR bronch* OR airway* OR nose* OR nasal* OR trach* OR larynx* OR alveol*) NEAR/2 (organoid* OR primary-human* OR cell-isolat* OR tissue-isolat*)) OR ((respirator* OR respiro* OR lung* OR pneumo* OR pulmonar* OR bronch* OR airway* OR nose* OR trach* OR larynx* OR alveol*) NEAR/2 (primar*) NEAR/2 (cell* OR cyte* OR culture* OR epithel*)) OR ((primar*) NEAR/2 (pneumocyt* OR AEC* OR HAE OR HAEs OR HAEC* OR NHBE* OR wdNHBE* OR BEC OR NHNC OR NEC OR hNEC)) OR PAEC* OR PBEC* OR HNEpC)) NOT ((animal* OR rat OR rats OR mouse OR mice OR murine OR dog OR dogs OR canine OR cat OR cats OR feline OR rabbit OR cow OR cows OR bovine OR rodent* OR sheep OR ovine OR pig OR swine OR porcine OR veterinar* OR chick* OR zebrafish* OR baboon* OR nonhuman* OR primate* OR cattle* OR goose OR geese OR duck OR macaque* OR avian* OR bird* OR fish*) NOT (human* OR patient* OR women OR woman OR men OR man))) AND LA=(English) AND DT=(Article OR Review) NOT TI=(case-report*)
